# Current Research and Applications of Starch-Based Biodegradable Films for Food Packaging

**DOI:** 10.3390/polym14061126

**Published:** 2022-03-11

**Authors:** Helen Onyeaka, KeChrist Obileke, Golden Makaka, Nwabunwanne Nwokolo

**Affiliations:** 1 School of Chemical Engineering, University of Birmingham, Edgbaston B15 2TT, UK; 2 Fort Hare Institute of Technology, Faculty of Science and Agriculture, University of Fort Hare, Alice 5700, South Africa; NNwokolo@ufh.ac.za; 3 Department of Physics, Faculty of Science and Agriculture, University of Fort Hare, Alice 5700, South Africa; GMakaka@ufh.ac.za

**Keywords:** starch, biodegradable film, food packaging, environmental impact, shelf life

## Abstract

The use of biodegradable packaging material as an alternative to conventional petrochemical-based polymers is based on the environmental issues associated with conventional materials. This review aims to update the existing knowledge regarding the application of starch-based biodegradable films for food packaging. From the review, it was evident that starch stands out among biopolymers due to its abundance and cost effectiveness. This review is the first of its kind, having reviewed over 100 articles/publications on starch-based biodegradable films, consolidating their current state of research and their applications for food packaging; therefore, this review provides an insight into the utilization of nanomaterials to improve the shelf life of packaging of food.

## 1. Introduction

**Figure 1:**
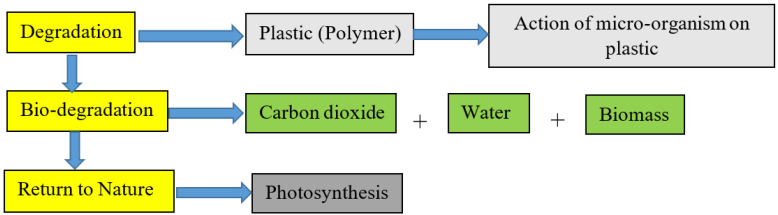
The process of biodegradation.

The use of plastic for food packaging in the food industry has shown an annual growth rate of 5% over recent decades. At present, plastic is recorded as the second most widely used material for food packaging [1]. This packaging process is essential as it prevents foods from being infected by microbes, thereby prolonging their shelf life. Real product shelf life is governed not only by microbiological control but also by chemical and physical control of products, connected to the maintenance of desirable sensory properties throughout storage. Food packaging is an essential part of the food industry sector. However, the food packaging sector is now in pursuit of lightweight biodegradable packaging for reducing materials use, waste, and transportation costs. The process of biodegradation (see Figure 1) is engineered by the activities of microorganisms present in the environment. During this process, microorganisms consume degradable plastics, thereby producing carbon dioxide, water, and biomass that is returned to nature by the bio cycle process [2].

However, there are certain limitations in the plastic degradation process, because it takes a long time, thus impacting negatively on the environment [3,4]. Some of the environmental impacts of plastics include the contamination of marine and land creatures during photodecomposition. Furthermore, particles of plastics are said to cause injury or death to marine life, thereby disrupting the ecosystem and food chain, leading to potential extinction. Moreover, some of the plastics are also non-recyclable, thus creating environmental burden. Despite the disadvantages associated with plastic disposal, studies have shown that the use of plastic packaging still represents about 37% of the total plastic demand [5,6]. To solve the elongated degradation problem associated with plastics, studies are ongoing for readily biodegradable materials for use as packaging materials for the food industry. Among all the biopolymers, starch-based films stand out because of the abundance and low cost of starch [7]. A biodegradable film derived from starch can become a primary packaging material made from biodegradable polymers and food-grade additives. According to the attestation made by Galus et al. [8], biodegradable films have been employed for the protection and extension of packaged food shelf life. The use of starch-based films in food products is backed by their inherent properties, which include biodegradability, edibility, and abundance. Moreover, biodegradable materials are said to have advantages over plastics in terms of environmental preservation. This is because biodegradable materials degrade after their deposal, creating a new agricultural product [4].

Recent review studies relating to starch-based biodegradable films have been focused on biodegradable polymer trends [9], materials for biodegradable food packaging [10], nanotechnology in food science [11,12], challenges and opportunities for starch-based materials [13], and extraction and sources of starch for biodegradable films [14,15,16,17]. These are interesting topics; however, no reviews have focused on the current state and applications of starch-based biodegradable films for food packaging—a gap in the literature which this study aimed to fill. Therefore, our review covers the topic of the production and processing of starch, the sources of starch, the current industrial applications of starch-based biodegradable films, the properties of starch, the addition of nanomaterials, the embedding of antimicrobial agents, and the evaluation of the shelf life of foods packaged with starch-based biodegradable films. Furthermore, the future perspectives of the present study are presented.

## 2. Starch as a Biodegradable Packaging Material

Starch is a good source of biodegradable material for food packaging, originating from wheat, corn, rice, and potatoes [18]. It is widely viewed as a sustainable substitute to plastics for food packaging. Moreover, various foods, such as fruits, vegetables, snacks, and dry products, can be packaged using starch as a biodegradable film [19]. The three ways starch can be used in producing biodegradable films are as follows: firstly, small amounts of starch can be used in the preparation of starch compositions with other plastics. The essence of this is to improve the biodegradability of traditional, oil-based starch materials. Secondly, the preparation of starch composites with the starch content comprising more than half of the mass. Thirdly, the use of extrusion, processing with mixtures of granular starch in biodegradable preparation processes [20]. The increase in the use of starch-based biodegradable films for food packaging is an outcome of its numerous advantages. These include their contribution to reducing fossil content, their lack of toxins, their origin being plant sources (renewable resources), their biodegradability and biocompatibility, the low cost and abundance of starch, their safety for consumption when used in food packaging, their reduced energy consumption, their role as an eco-friendly disposal solution, and the absence of a net increase in CO_2_ in the global ecosystem. Despite these advantages and benefits, disadvantages include poor mechanical properties, low water stability, high moisture sensitivity, presence of a poor moisture barrier because of strong hydrophilic behaviour, their brittle behaviour at room temperature, and their high moisture content [1]. To overcome these disadvantages of using starch as a biodegradable material, a thermoplastic starch matrix could be filled with nanofillers, thus improving these properties. In a study by Nafchi et al. [21], the addition of nanoscale particles enhanced the mechanical and barrier properties of starch. Montmorillonite (MMT) nano-clay has been recommended as promising nanoscale filler for biodegradable packaging. Hence, the use of MMT in food packaging can be attributed to its reduced cost, high stability, and high level of effectiveness. [22]. More information on MMT nanoclay is detailed in the present review. Studies have shown that the properties of starch-based films are responsible for its rigidity and reduced flexibility. Hence, other polymers could be used as additive compounds [1]. Different sources of biopolymers, such as polysaccharides, proteins, and lipids, can act as biodegradable films [23].

Starch is mainly sourced from plants but can also originate from roots, tubers, cereals, and legumes. Considering its inherent biodegradability, abundance, and annual renewability, starch is a promising natural polymer. Interestingly, previous studies have revealed that starch contains two kinds of microstructures: linear and branched. Hence, it is regarded as a heterogeneous material. The linear structure is known as the amylose (crystallizable form of starch made up of long unbranched polysaccharide chains), while the branched structure is called the amylopectin (non-crystallizable form of starch with branched polysaccharide chain).

**Figure 2:**
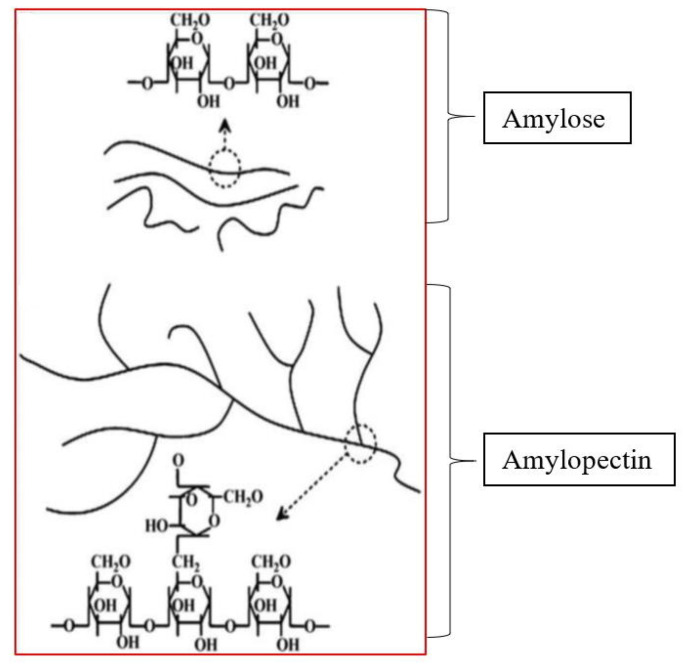
Chemical representation of two kinds of microstructures in starch Modified from Jiang et al. [13].

Figure 2 shows a chemical representation of amylose and amylopectin starches.

In the production of starch-based films, plasticizers are necessary to curb the effect of brittleness due to polymer chain interactions. Plasticizers tend to improve mechanical properties, reduce the tension of deformation, hardness, density, and viscosity, and increase polymer chain flexibility and resistance to fractures [9,24]. We have established the fact that starch-based biodegradable films are promising substitutes for conventional or synthetic films for food packaging. These have been studied in terms of their mechanical and optical properties. At this point, the stability of biodegradable films is determined by the evaluation of the zeta potential, regarding the charges of the polymer chain that affect aggregation and consequently the microstructural network [25,26,27]. Generally, biodegradable films are mostly characterized from a mechanical and optical point of view. These are relevant for food packaging, as such characteristics help to increase shelf life and protect against incident light. However, less attention has been given to characterizing the biodegradable films forming dispersion through the study of rheology properties and stability [28]. In [29,30,31], Turbiscan equipment was used to investigate the destabilization phenomena of inorganic phase change material; it was later observed and revealed that this equipment is also promising in other dispersion-based applications.

### 2.1. Sources of Starch

Starch comes from corn, wheat, potato, cassava, and rice, as shown in Table 1 These starch sources contain 60–70% amylopectin and 30–40% amylose [32]. Table 2 presents the different sources of starch and their properties.

### 2.2. Effects of Starch as a Biodegradable Film

The previous section established that starch is a good biodegradable material for food packaging, particularly for dry products, fruits, and vegetables. In this section, the effects of these starch-based films as a substitute for plastic are discussed (See Table 3).

### 2.3. Production and Processing of Starch

Locally, starches are obtained from numerous sources, which include cereals, tubers, and roots. Recently, there has been an increase in starch production due to an increase in demand. In terms of production capacity, China and Brazil are topping the list, accounting for about 10% increase per year, and other countries have shown around 1–2% growth per year [48]. From the data of the European starch industry association, North America and East Asia are leading in the continent, with high production capacities of about 33% each. This is followed by Europe and Southeast Asia, with 18% and 11%, respectively, and finally South America, having 5% as the continent with the lowest production. Globally, 60% of the world market is involved in starch production, while confectionery, drinks, and processed foods account for 31% and 29%, respectively [48]. Extrusion (melting–solidification) is a known method used for the processing of starch. It involves the starch swelling, loss of birefringence, melting, and solubilisation of starch granules [49,50].

During the extrusion processing of starch, most amylose remains in amylopectin due to lower water content. Amylopectin is known to have a short-branched chain, which can be torn apart during gelatinization. This is a result of the formation of a double-helical crystalline structure. Gelatinization occurs at a lower water level due to the shear forces, which affect the starch granules, thereby permitting water transfer into the interior molecules [51].

Studies have been conducted regarding starch processing by extrusion, revealing that a decrease in the rate of amylopectin fragmentation with a decrease in screw speed results in an increase in temperature of around 121–177 °C [51,52,53]. According to Carvalho et al. [54], starch degradation is reduced using glycerol under shear stress. However, without shear stress, Olkku et al. [55] revealed that the main factors responsible for monitoring are water content and temperature. Any temperature below 50 °C is affected by van der Waal forces or hydrogen bonding during the stabilization of starch granules and their molecule constituents. This is attributed to intact crystalline components. The heating of starch granules above their gelatinization temperature results in greater swelling and dissolution of crystalline. This is because of the attachment of the disruption of hydrogen bonding and water molecules to the hydroxyl group of the starch molecules [56,57].

### 2.4. Extraction of Starch

Starch extraction is a key step in the production of starch-based biodegradable films for food packaging. The extraction of starch from yam and taro was conducted by Andrade et al. [58]. Using an industrial blender, the sample was ground into a paste by adding 3 L of distilled water. The obtained fluid-like paste was sieved using an 80-mesh sieve. Afterwards, the volume of the residual was measured, which doubled the amount of water that was added. The grinding process was repeated and sieved afterwards using a 200-mesh sieve. The resulting paste was kept for 24 h before the removal of the supernatant. The supernatant was separated from the starch precipitate and then dried in a forced-air dryer at a temperature of 40 °C after centrifugation at 3000 rpm. Similarly, Altemimi [15] explored a study in which starch was extracted from yellow-skin potatoes. The methodology of the study involved the peeling, slicing, and chopping of the potatoes into small chunks. After that, water was added to the chopped potatoes. The study employed the method of centrifugation as the extraction process, using speeds of 1000, 2000, and 4000 rpm, occurring for 5, 10, and 15 min, respectively. For the purpose of obtaining the wet starch, Whatman no. 1 and supernatant was used. This was aired for 5 h at room temperature to dry before crushing the dry starch into a fine powder, a process used in extracting starch in large quantities, especially in developed countries; *Ipomoea batatas* is a major source of extraction, particularly in an environment where 95% of the world’s food production depends on starch [15].

In a study by Agyepong and Barimah [14], starch was extracted from cassava varieties. The cassava was sorted, peeled, and cut into 2–3 cm^3^ chunks. After that, it was washed using distilled water and refrigerated. With the aid of a double-screw-waring blender, the 100 g diced cassava pulp was blended at a low speed for 1 min. The chilling process was employed to minimise the starch gelatinization during blending. The formed cassava mash was dissolved after being transferred into a conical flask of 600 mL, to which was added 100 mL of distilled water.

In a study by Tejavathi et al. [59], starch was extracted following the procedure conducted by Moorthy [60]. The fresh rhizomatous rootstocks were peeled and washed, thereafter cut into small sizes. The sample of the starch was homogenized separately using an ammonia solution of 0.03 M in a laboratory blender. The formed pulp was filtered through a fine muslin cloth after a time of 30 min had elapsed. The residue formed during this process was retained on the muslin and was homogenized in an ammonia solution of 0.03 M. After repeating the process about 5 times, the supernatant layer was thrown away, and the sediment starch was used. The obtained starch was characterised after successful extraction.

The ethanol method was seen as one of the methods used to extract starch, as confirmed in a study by Ramil et al. [61]. In the study, starch from microalgae biomass was extracted, characterized, and compared with commercial corn starch. The authors reported that starch extraction using ethanol removed substances such as pigments that affect the value of the starch. This was revealed after the extraction of microalgae biomass 3 times with 80% ethanol by vortex. The process took a minute and later was heated at 95 °C for 10 min. As observed from the above studies, starch can be extracted using different mechanisms.

Having briefly discussed the extraction of starch, the extracted starch is used to form starch-based biodegradable materials by the application of heat to form a filmogenic solution. It is interesting to note that starch with high amylose content is preferred for this purpose because of the bigger crystalline domain, which gives greater mechanical resistance [62]. However, with the increase in temperature, the extracted starch vibrates intensely, thereby breaking the intermolecular bonds and establishing hydrogen bonds with water. During this process, a decrease in the number and size of crystalline regions occurs. Moreover, the viscosity of the solution increases because of the swelling, and the starch molecules stick to each other with the agitation, acquiring a gelatinous aspect [63]. Rodrigues et al. [64] and Patkar et al. [62] detailed experimental studies of the formation of extracted starch to produce starch-based biodegradable materials. The starch-based biodegradable materials formed from the extracted starch are subjected to tests, such as the water solubility test, the biodegradability test, and the tensile strength, peak load, and break elongation tests.

### 2.5. Previous Reviews on the Application of Starch-Based Biodegradable Material

Most of the applications of starch-based biodegradable material in the literature have been carried out at a laboratory scale rather than at an industrial scale. In a study by Molaee Aghaee et al. [65], the researchers packaged a chicken filet using chitosan films containing garlic basic oil amid capacity at a refrigeration temperature. Different levels (0, 0.5, 1.0, and 2.0%) of garlic basic oil was added to the chitosan film arrangement. The chicken filet was examined chemically on days 2, 4, 7, and 10, and it was revealed that the sample films showed a lower pH, which was attributed to unstable nitrogen, thiobarbituric-acid-reactive substances, and peroxide. Thus, the study concluded that chemical deterioration components were prevented from developing as a result of the chitosan films used for packaging the chicken.

In another study, the effect of starch protein films on *Lactobacillus rhamnous* was studied by Soukoulis et al. [66]. The scope of the study focused on compositional, physicochemical, and auxiliary characterization. Starch from local rice and corn was used in the study. In addition to the starch, bovine skin gelatine, sodium caseinate, and soy protein also were also used for the creation of the starch film through probiotics. The study’s findings showed an increment in the practicality of *L. rhamnosus* by 3–7-fold around the nearness of protein with sodium caseinate. The study was concluded by calculating the shelf life of the films. The results showed that, in accordance with the framework of the premise of 6 log practical CFU/g, the measure was extended at 27–96 days and 15–24 days at ice chest or room temperature, respectively.

A comparison study focusing on the main and physical features of chitosan and altered starches of edible films was examined by Garcia et al. [67]. The films’ arrangement was made using a casting strategy that utilises chitosan, waxy, oxidized, and acetylated corn, and their respective blends. Their studies revealed a clearing out of chitosan with a less positive charge, due to the connections between the acetyl bunches, acetylated with carboxyl, and the amino bunches of chitosan. It was also seen that the positive charge diminished because of the interaction with the amino bunches of chitosan, which affected the antimicrobial action. The study concluded by mentioning that the adjustment of the starch affects chitosan, thereby driving the diverse of the films’ features.

The aim of a study conducted by Gomes et al. [68] was to characterize edible films of *S. burchelli* phosphate starches, and observe the development of coasting and its application to cherry tomatoes through post-harvest conservation. The following measurements were conducted during the study: the thickness of the film, the solubility in water, and the permeability by water vapour. The methodology of the study involved comparing the conservation of the cherry tomatoes with and without coverage at the following conditions: time—21 days; temperature—10 ± 2 °C; relative humidity—80 ± 5%. The study revealed that factors such as reduction in water solubility, increase in permeability, and characteristics of the films were usually affected by the concentration of the glycerol and the type of starch used. In addition, it was reported that fruits with an edible coating showed a greater permeability by water vapour, especially for the conservation of cherry tomatoes. This was attributed to the gradual decrease in the film during storage compared with the control from an experimental point of view. The study’s objective was successfully established, proving that the concentration of glycerol affects consumable films and should allow for ideal post-harvest use.

Adjouman et al. [69] conducted a study on the water vapour porousness (WVP) of edible films on 4 g cassava starch from Cote d’Ivoire, focusing on the response of the starch in respect to the effect of glycerol (25–30%), shelled nut oil (5–10%), and soybean lecithin (0–5%), all in w/w. Temperature (25 °C) and relative humidity (75%) were monitored and obtained to determine the water vapour porousness. The findings from the study show that glycerol and shelled nut oil increased the WVP, while the soybean lecithin did not affect the WVP. In addition, the following results were obtained for the WVP: glycerol—25%; shelled nut oil—5%; soybean lecithin—5%. In Cote d’Ivore, starch from cassava is said to be a promising starch for nourishment bundling.

Xiaoyang et al. [70] recommended the use of iron yam and maize starch flavoured with fundamental or essential lemon oil (plasticization). The following parameters were examined: physical change, microcosmic features, and antimicrobial of the starches. The study reported diminished dampness substances, water vapour porousness, solvency, and malleability quality. These factors reported were a result of the presence of the lemon fundamental oil used in the study. In conclusion, the recommendation was proven as iron yam/maize starch can be used to nourish flavour for the packaging material. This is because of their physical and antimicrobial characteristics. To conclude this section, in order to make starch useful as a packaging material, it certainly seems to require a lot of support through the addition of many other non-starch materials. This addition is highly recommended.

### 2.6. Application of Starch-Based Nanomaterials

To keep foods from being infected by fungi and bacteria and for the purpose of long-term storage, nanotechnology is essential [1]. Additionally, by integrating nanomaterials into the food industry, food quality and safety can be further improved [71]. According to Singh et al. [72] and Gupta et al. [73], nanomaterials are characterised by three major properties. These include unique properties (high ratio of surface to volume), physiochemical properties (solubility, optical, magnetic, etc.), and thermodynamic properties. Furthermore, materials used in nanotechnology are non-toxic [74], and at high temperatures and pressures, they are stable [75,76]. These properties have contributed to the extension of shelf life and newness of packaged products. Kuswandi and Moradi [77] recommended using different functional nanomaterials to improve the quality of materials used for food packaging, which can prolong the life span of the packaged food and its safety.

According to Joye et al. [78], Khare et al. [79], and Yoksan and Chirachanchai [80], nanotechnology is formed by the combination of nanoparticles to form nanofilms. Nanofilms decrease gas permeability, thereby reducing harmful concentrations of gases such as carbon dioxide (CO_2_) or oxygen (O_2_). These gases negatively impact the shelf life of the food products and act as an obstacle that hinders microorganism activities. This acts as one of the advantages of nanomaterials in food packaging. According to Brody [81], Joye et al. [78], Khare et al. [79], and Yoksan and Chirachanchai [80], the use of nanomaterials can lead to a decrease in oxygen and carbon dioxide permeation of up to 80–90%. From this study, the authors pointed out that most food products are oxygen-sensitive. Hence, a packaging gas barrier seems important for the safe horticultural production of most product types. Carbon dioxide is generally not detrimental to foods and is used in gas-flushing-modified atmospheric packaging (MAP) above 20% to selectivity impact aerobics and psychotropic microorganisms. This makes carbon dioxide gas barrier properties in packaging materials very important.

**Figure 3:**
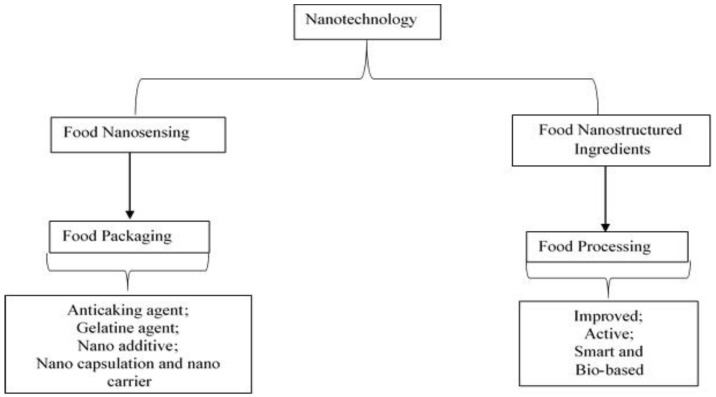
Classification of nanotechnology and its applications (modified from Primozic et al. [11]).

Interestingly, nanotechnology can be classified into food nanosensing and nanostructured food ingredients [82]. Food nanosensing focuses on improving food quality and their safety, while nanostructured food ingredients can be used in a wide range of food processing and packaging applications. Both categories are presented in Figure 3.

Having established the fact that the use of nanomaterials or nanotechnology in food packaging improves food quality and safety, it would be interesting to briefly review these materials in starch-based films (starch-based nanomaterials). Material for food packaging using starch-based biodegradable films must be durable. To accomplish this, starch-based nanomaterials are necessary. One main function of the starch-based nanomaterial is to reduce the weakness of the natural polymer in starch [1]. Starch is a promising biopolymer for food packaging, which is affected by water sensitivity and brittleness [82]. The mechanical, UV, and water properties of starch material can be improved by adding nanoparticles [83].

Considering a few studies on starch-based nanotechnology and its effects, the utilization of different concentrations of graphene oxide and its response to starch–graphene oxide composite film was studied by Wu et al. [84]. The study revealed that the addition of graphene oxide greatly impacted the packaging material in terms of mechanical properties and water permeability. In a study by Aqlil et al. [85], an investigation into the employment of a graphene-oxide-filled starch/lignin polymer with bio–nanocomposite was conducted. The investigation showed that graphene oxide had a strong influence on the strength of the material and has the potential to reduce water vapour permeability and moisture characteristic of the starch-based film.

Furthermore, the incorporation of the starch film with multi-walled carbon nanotubes was carried out by Shahbazi et al. [86]. From the findings, there was an improvement with nanotube inclusion because of the hydrophobic characteristics of the film. A study by Oleyaei et al. [87] estimated the influence of the thermal, mechanical, and barrier features of titanium dioxide and montmorillonite on potato starch. From this, it was reported that there was an improvement in the tensile strength, melting point, and elongation break as a result of the addition of montmorillonite (MMT) and TiO_2_.

Additionally, through the hydrolysis method with pullulans, corn starch films were prepared with the aid of taro starch nanoparticles (TSNP). The methodology decreases the vapour permeability and increases the opacity because of the addition of TSNPs to the starch source (corn). From the experiment, Dai [88] reported that a concentration of TSNP in the starch film of 10% (w/w) generated a tensile strength of 2.87 MPa. This finding improved the thermal properties of the starch film.

In another study based on the reinforcement of rice-starch-based film with starch nanocrystal, conducted by Piyada et al. [89], the tensile strength increased as the elongation break decreased. Additionally, a starch nanocrystal of lower content affected (increased) the rice starch film in terms of the crystalline peak structure. Similarly, a study conducted by Tian and Xu [90] supported Piyada et al. [89], where the former confirmed that tensile strength, and Young’s modulus increased with a slight decrease in elongation break. This was proven in an experiment with glycerol-plasticized soy protein plastics incorporated with citric-acid-modified starch nanoparticles of an average size of 82 nm.

Adding nanoscale particles improved the crystallization kinetic, crystalline morphology, crystal form, and crystalline size of the starch. Interestingly, MMT nanoclays are promising fillers for biodegradable packaging. This is because they are less expensive, and they are effective and stable [23]. The MMT nanoclay is characterised by a thickness of 1 nm and average lateral dimensions ranging between few tenths of a µm to several µm. This required dimension possessed by the MMT nanofiller is recommended because of the high surface of the nanosized fillers, which depends on the nanocomposites. This results in a large interface between the matrix or biopolymer and the nanofiller. One major property of the presence of a large interface, as mentioned by McGlashan and Halley [91], is its ability to improve the biocomposite’s properties (physical, thermal, and water barrier). This is necessary for the food sector, where biocomposites are usually developed to exhibit the properties mentioned earlier, which are needed during food processing and preservation.

## 3. Evaluation of the Shelf Life of Foods

Various calls from consumers have brought about the need for extended shelf life in the food industry. There is a need to meet the desire for food quality to be highly maintained throughout purchase and consumption. By definition, the shelf life of food deals with the safety of food products to retain the desired sensory, chemical, physical, and microbiological characteristics [92]. For extended shelf life to exist, food must be processed and stored for purchase and consumption while maintaining these characteristics [93]. To properly evaluate the shelf life of food, a good understanding of highly perishable, semi-perishable, and highly stable food products is required [94] (see Table 4). Highly perishable foods (fresh meat, vegetables, milk) are products that are deteriorated by the action of enzymes and microorganisms. Semi-perishable foods (cheese, bakery products, and smoked meats) contain natural inhibitors and receive minimal treatment in terms of preservation. Highly stable foods (dried, frozen, and canned foods) have been subjected to a thermal process and are maintained in a specific condition. Table 4 presents the duration of evaluation of shelf life.

Compositional and environmental factors can influence the evaluation of a product’s shelf life [9]. Compositional factors deal with the properties (water activity, pH value, total acidity, and food composition) of the final product, while environmental factors focus on the stages which the final product passed through as it moved through the food chain. Such factors include temperature, relative humidity, and the time–temperature profile. Series of reactions (biochemical and physiochemical) are needed to understand the mechanisms that contribute to the spoilage of food products, while reactions that deal with chemical and enzymatic activity and moisture or vapour migration reflect food deterioration [95]. Therefore, developing active materials for packaging and reducing food spoilage and waste is necessary to improve food shelf life and safety [11]. Furthermore, materials that enhance the shelf life of foods and their properties are presented in Table 5.

According to Primozic et al. [11], these mediums or agents (see Table 5) could act as conventional non-degradable packaging, used in conjunction with biodegradable compounds. Interestingly, in new-generation active packaging—such as emitting sachets or coating antimicrobial agents—the antioxidants flavour and preservation functions help improve the quality and safety of foods.

### Types of Shelf Life Evaluation and Design

Not many studies have been carried out regarding the shelf life evaluation and design. According to Kilcast and Subramaniam [102], shelf life evaluation is divided into three types: static, accelerated, and abuse evaluation. Static evaluation focuses on storing food products at a given set of conditions. Static evaluation is associated with its high costs of implementation and long-term duration before any changes can be observed. For accelerated evaluation, food product storage is based on environmental factors (temperature and relative humidity). These factors do not usually alter the anticipated path that influences food products’ shelf life, because of the provision of kinetic data that this type of evaluation offers. The recycling of food products using environmental variables is referred to as abuse evaluation. With abuse evaluation, both package and product are assessed as a unit [102].

Microbial safety should be considered during the design of shelf life. For instance, in the process of frozen storage, biochemical changes take place in the frozen products. To avoid this, microbiological testing results are necessary before samples are tested for other qualities. For accelerated evaluation, the temperature differences (5–10 °C) vary. In this case, high-temperature storage methods are avoided [103]. Considering the basic design sampling of the shelf life evaluation, the frequency of the sample should increase or decrease appropriately. Hence, the cost of this type of design seems to be high due to the panel’s repeated training before each evaluation. This process is possible if the sample is evaluated for sensory panels. The reverse design sampling is initiated by the collection of the sample following the sampling plan. During this type of design, the quality sample’s stability is ensured (freezing or refrigerator) by maintaining the sample in controlled conditions. Basic design sampling is advantageous because of its low cost in evaluating sensory characteristics; however, the rate (faster or slower) at which the sample changes might be a problem [103].

## 4. Challenges Facing Starch-Based Biodegradable Films for Food Packaging

Before concluding the study and recommending areas for future studies, it is necessary to briefly look at the major challenges faced by starch-based biodegradable films for food packaging. Although the disadvantages have been mentioned, poor mechanical behaviour and high water vapour permeability (mass of water vapour transmitted through a film area within a defined time) are the main challenges or drawbacks associated with starch-based biodegradable films. The poor mechanical behaviours are determined by tensile tests and they include the tensile strength, the strain at break, and the elasticity modulus [104]. In relation to high water vapour permeability, the issue of hydrophilicity is concerning. Hydrophilicity is one of the properties derived from the polar characteristics of starch hydroxyl groups and is regarded as an important factor affecting starch-based biodegradable films for food packaging. One of the methods to reduce the hydrophilicity of starch-based biodegradable films is to combine them with lipids. To achieve this, emulsifiers such as polydimethylsiloxane or commercial polysorbate surfactants are applied. According to Cao and Song, [105], Evagelho et al. [106], Kang and Song [107], and Hasan et al. [108], the emulsifier interacts with the starch network. The hydrophobic nature prevents interaction between the starch and water. A more organized network arrangement with increased crystallinity can be used to reduce the hydrophilicity of starch-based biodegradable films. This is possible because of the higher molecular density. Thus, higher solubility has the potential to speed up the biodegradability process and facilitate waste management after disposal [109,110]. Finally, a discussion of the challenges facing starch-based biodegradable films for food packaging would not be complete without talking about the processability of the starch-based films. This process is more difficult to control than it is in the case of conventional plastics. Although the processing of starch has been discussed earlier in Section 2.4, it was shown that the amount of water needed emphasises the differences experienced in the techniques used for starch processing. The use of water and high temperature came about as a result of the drawbacks/challenges encountered during the processing of starch. For this reason, there is a need for traditional processing techniques, thereby controlling the process conditions and judicious incorporation of specific additives which are commonly used [104].

## 5. Conclusions and Outlook

This review succinctly accounted for the current state of research and applications of starch-based biodegradable films for food packaging. The study was motivated by the necessity of finding a substitute (biodegradable starch film) for the conventional synthetic plastic currently in use. Although biodegradable starch films are associated with poor properties, the utilisation of nanomaterials tends to enhance the brittleness and physical behaviour of the films for food packaging. While nanotechnology offers various potentials in food packaging—as a result of its functions from bio-based packaging to smart packaging in the food sector, as well as improving food quality and safety—further study is recommended to ascertain the advantages and disadvantages of using nanotechnology in food packaging materials. The issue of moisture sensitivity was identified as a limitation in the use of starch-based materials for food packaging. As further observed, the tensile strength and adequate water vapour permeability of the films can be attributed to the hydrophilic nature of starch. This makes starch-based films susceptible to moisture uptake. Hence, to enhance the resistance of starch-based materials to moisture and mechanical properties, various blending and composting techniques are required, such as coating (acrylate-epoxidized soybean oil). Addition of acrylate-epoxidized soybean oil reduces moisture sensitivity and increases the gas permeability of the starch-based films. To improve the surface adhesion or bonding between starch and acrylate-epoxidized soybean oil coating, polyethylenimine (PEI) is recommended. With regards to the evaluation of shelf life, food materials packed using starch-based films provide a platform for microbial spoilage of stored foods. To avoid this, it is recommended that the use of essential oils with antimicrobial and antibacterial potentials or properties should be employed. This provides a remedy to such limitations.

Further work should focus on improving the performance of starch-based films by reducing their moisture sensitivity, while considering the balance between different chemical treatments for reducing moisture sensitivity and biodegradability. During the review, it was observed that studies regarding starch-based materials in other applications, such as in use as fertilizers and in water treatments, have a lot of potential and interest. Therefore, there is a need for researchers and academics to examine this direction.

## Figures and Tables

**Table 1:** Sources of starches and their types.

Types of Starch	Sources	References
Corn	Soya flour, cassava starch, and corn	[33]
Potato	Rice flour, potato, white rice flour, soya flour, and egg powder	[34]
Cassava	Sorghum flour	[35]
Wheat	D-glucose, bakery yeast, locust bean gum, and wheat starch	[36]
Tapioca	Corn flour, soya bean flour, and cornflour	[37]

**Table 2:** Various sources of starch and their properties [9].

Biological Source	Geographical Source	Macroscopically Characters	Microscopy of Some Starch	Chemical Constituents
Starch consists of polysaccharides granules from the grains of Maize *Zea mays* L., rice *Orza sativa* L., wheat *Triticum aestivum*, or from the tubers of the potato *Solarium tuberosum* L.	Starch is produced in tropical and subtropical countries, such as Argentina, the USA, China, and India. However, Japan is regarded as the main starch-producing country globally.	It is found in irregular, angular masses or white powder. Insoluble in cold water and forms colloidal solution on boiling. Starch solution becomes a translucent jelly after cooling.	Germs are continuously separated from the suspension by liquid cyclones and used in the preparation of germ oils. The germs oil is characterized to be rich in vitamins.	Starch contains a mixture of two polysaccharides—80% amylopectin and 20% amylose. Amylopectin is insoluble in water, while amylose is soluble in water.

**Table 3:** Properties of starch-based biodegradable films for food packaging.

Properties	Description of the Properties
Structural properties	To examine the chemical structure and composition of packaging material, atomic force microscopy and Fourier transform infrared (FR-IR) spectroscopy were used [38]. Starch and PVA films exhibit homogenous and smooth surfaces. One factor influencing the structural properties is phase separation, which only occurs in the amylopectin type of starch. The phase separation is due to the amount of starch and phosphate groups. Recommendation indicated that the thickness of biodegradable packaging material should be less than 254 µm [39].
Solubility properties	The solubility properties are directly proportional to the hydrophilic nature of polymers. For example, starch film and PVA solubility are reported to be 0.208 g _dissolved_/g _dry films_ and 0.19 g _dissolved_/g _dry films,_ respectively [40]. The recommendation of aqueous medium for packaging and storage is its low solubility values, which shows good stability [34].
Mechanical properties	Biodegradable polylactic films exhibit poor mechanical properties compared with polylactic petroleum films [41,42,43]. Mechanical properties are associated with the crystallinity of polymer and content of amylose [44], the weight of properties, additive concentration, and distribution. The high tensile strength and elongation break experience in starch films result from the low molecular weight.
Optical properties	The decolourisation and deterioration of packaged food products are caused by overexposure to ultraviolet (UV) and visible radiations. To carry out quality control in packaged food products, transparency and UV screening are essential. Based on Vaezi et al. [45] study, nanocomposites increase the non-transparency of starch films, which suggests that nanoparticles are UV blockers, thereby minimizing the passage of light.
Permeability properties	The polymer matrix exhibits effective permeability of gases, which increases the shelf life of food products [46]. The shelf life and freshness of food are directly proportional to water transfer between the product and its surroundings. Hence, the main function of packaging has to deal with reducing the transfer of water. According to Yu et al. [47], silica nanoparticles’ presence in biodegradable films decreases moisture permeability.

**Table 4:** Duration of evaluation of shelf life.

Classification of Foods	Duration of Measurement
Highly perishable	Every day
Semi-perishable	Every two weeks
Highly stable	Every week or monthly

**Table 5:** Enhancement of food packaging (extract from Primozic et al. [11]).

Types of Food Packaging	Characteristics	Medium/Agent	References
Oxygen scavengers	Oxidation of fat is prevented and avoided.	Metallic iron powder; organic (ascorbic acid); inorganic (ZnO); polymer- and enzyme-based agents (glucose).	[96]
Ethylene scavengers	Reduces the ripening of fruit and vegetables.	PdCl_2_; Pd-impregnated zeolite; polyvinyl chloride film containing ZnO nanoparticles; inorganic (silica gel); inorganic (xylitol, fructose).	[97,98]
Moisture absorber	Reduces the growth of microorganisms.	Polymer-based agents (starch).	[99]
Carbon dioxide	Inhibit food spoilage by the action of microorganism.	Citric acid, bicarbonate and ascorbate, and sodium.	[100,101]
